# Home Chalets for Tubercular Patients

**Published:** 1910-01-22

**Authors:** Stanley H. Bates

**Affiliations:** Dartmoor, Maldon Road, Wallington, Surrey


### Editor's Letter-Box.

### HOME CHALETS FOR TUBERCULAR PATIENTS.

*To the Editor of* The Hospital.

Sir,—In your issue of the 15th inst. I notice a reference, under the above heading, to my booklet entitled, "Open-air at Home—Sanatorium Treatment Continued."

I should be much obliged if you could find room in your columns to make what I think you will agree is a very necessary correction. Speaking of the garden shelter I used after returning from the sanatorium, the writer says: "He began to occupy it in September 1906, and the shelter has ever since been his home. He declares he has enjoyed life in it as much in winter as in summer, *and has never been indoors more than* 10 *minutes in any* 24 *hours.*"

A reference to my introduction (p. 18) will show clearly that the strict discipline described in the last sentence (which I have placed in italics) was only maintained during the first year of my home treatment, and that "these rigid rules were relaxed in the following summer," on my partial return to my occupation.

I write to ask you to make this correction because, in writing my experiences, I endeavoured to confine myself to a plain statement of facts without any over-estimates, and was rewarded by Sir James Crichton-Browne's statement in his Prefatory Note, that the book was free from exaggerations. The impression of three years' strict treatment which the statement in your article conveys, must lead to some measure of incredulity on the part of your readers, and, further, detracts from the value of garden-shelter treatment, in that such a long period should seem necessary.

I am in cordial agreement with the suggestions contained in your article.

Yours faithfully,

Stanley H. Bates.

Dartmoor, Maldon Road, Wallington, Surrey,

